# Effectiveness of Different Telerehabilitation Strategies on Pain and Physical Function in Patients With Knee Osteoarthritis: Systematic Review and Meta-Analysis

**DOI:** 10.2196/40735

**Published:** 2023-12-04

**Authors:** Wu Xiang, Jun-Yu Wang, Bing-jin Ji, Li-Jun Li, Han Xiang

**Affiliations:** 1 Department of Rehabilitation Beibei Traditional Chinese Medical Hospital Chongqing China; 2 Department of Rehabilitation Medicine Shanghai Fourth People’s Hospital Affiliated to Tongji University School of Medicine Shanghai China; 3 Department of Radiology Daping Hospital Army Medical University Chongqing China

**Keywords:** telerehabilitation, telemedicine, knee osteoarthritis, pain, physical function, systematic review, meta-analysis

## Abstract

**Background:**

Knee osteoarthritis (OA) is a chronic, degenerative bone and joint disease. It can lead to major pressure to the quality of life and mental health of patients, and also brings a serious economic burden to society. However, it is difficult for patients with knee OA to access rehabilitation when discharging from the hospital. Internet-based rehabilitation is one of the promising telemedicine strategies for the improvement of knee OA, but the effect of different telerehabilitation strategies on knee OA is not clear.

**Objective:**

The aim of this systematic review and meta-analysis was to identify telerehabilitation strategies attributing to the improvement of pain and physical function outcomes in patients with knee OA.

**Methods:**

We reviewed and analyzed telerehabilitation strategies from randomized controlled trials (RCTs) comparing telerehabilitation with conventional treatment or usual care. For each strategy, we examined whether RCTs that applied the telerehabilitation strategy resulted in a significant improvement in pain or physical function compared with conventional treatment or usual care.

**Results:**

We included 6 RCTs (n=734) incorporating 8 different telerehabilitation strategies. The duration of the interventions ranged from 1 to 48 weeks, and sample sizes ranged from 20 to 350 patients. The results showed that RCTs that provided telerehabilitation were found to be more effective than conventional treatments for improving pain (*P*=.003; standardized mean difference [SMD] –0.21, 95% CI –0.35 to –0.07), but not physical function (*P*=.24; SMD –0.09, 95% CI –0.25 to 0.06). Furthermore, this systematic review and meta-analysis indicated that there is no significant correlation between different telerehabilitation strategies and the pain and physical function of patients with knee OA.

**Conclusions:**

This systematic review and meta-analysis showed that telerehabilitation programs could relieve pain but not improve physical function for patients with knee OA. These results indicated that telerehabilitation is beneficial for the implementation of home rehabilitation exercises for patients with knee OA, thereby reducing the economic burden of health. However, there were limitations in terms of the number of search results and the number of studies that were eligible for this review and meta-analysis. Therefore, the results need to be interpreted with caution, and more high-quality studies with large samples are needed to focus on the long-term outcomes of telerehabilitation for patients with knee OA to address this limitation.

## Introduction

Osteoarthritis (OA) is a chronic degenerative joint disease involving cartilage destruction, synovial inflammation, osteophyte formation, and subchondral bone remodeling [1,2]. There are several clinical symptoms associated with this syndrome, including joint pain, stiffness, swelling, deformity, and dysfunction. Epidemiological studies show that approximately 250 million people suffer from OA worldwide, with knee OA being the most common [3,4]. The incidence of knee OA is continuously growing with increasing obesity and the prolonged life expectancy of patients. As some studies have indicated, approximately 10% of men and 13% of women aged 60 years or older have characteristic knee OA [5]. For patients older than 70 years, the incidence increases to 40% [5].

The etiology of knee OA may be the result of the interaction of multiple factors, including age, obesity, trauma, increased joint weight bearing, and decreased joint stability. The degeneration of human joint tissue with aging may ultimately lead to cartilage loss and osteoarthritic changes [6]. Meanwhile, obesity can increase the mechanical pressure on the knee joint [7], aggravate cartilage damage, and cause abnormal bone metabolism and remodeling responses, leading to increased knee joint load [8]. In addition, trauma can directly damage knee joints, especially repetitive exercise-induced joint injury (eg, squatting and kneeling in older people). Occupations that require squatting or kneeling for more than 2 hours a day were associated with a significantly increased risk of moderate to severe knee OA [9]. Abnormal gait can lead to increased joint load bearing and decreased joint stability. One study has shown that women are twice as likely as men to suffer from knee OA, which may be related to heel height. Heel height can significantly affect knee kinematics and dynamics during walking, and walking in high heels can increase knee extension torque, thereby increasing knee joint load [10]. It has been reported that mechanical [11,12], inflammatory [13-16], metabolic [17], and cellular factors [18,19] as well as the balance between the destruction of joints and their repair are also associated with knee OA. These studies showed that knee OA poses an enormous threat to global health because of the high incidence rate, lack of effective efficacious pharmacotherapies, and poor prognosis. Despite enormous advances in modern medicine, chronic pain and impaired physical function are still the most common functional impairments in patients with knee OA [20,21], and it is difficult for them to access rehabilitation when discharging from the hospital [22]. Therefore, it is necessary to develop new therapeutic approaches to solve this disease.

During the COVID-19 pandemic, home telerehabilitation became a widely used strategy for knee OA rehabilitation in the patient’s home guided remotely by the therapist using telecommunication technology [23-26]. Several randomized controlled trials (RCTs) suggest that the pain and physical function of knee OA are improved by telerehabilitation [27-32]; however, the outcomes from individual RCTs are heterogeneous [28,33]. Owing to these inconsistent research findings, the use of telerehabilitation in knee OA has been questioned. Otherwise, several studies have evaluated specific approaches to telerehabilitation for knee OA, including mobile health [34], structured telephony [31,35], education [36], medication [37], physical activity [31], and physiotherapy support [28,33,38,39]. These studies provide a reference into the effectiveness of different interventions, but do not explain results involving different telerehabilitation interventions.

In view of the growing number of RCTs of different telerehabilitation strategies for treating knee OA, we conducted a systematic review and meta-analysis of the available evidence to inform clinical therapy. Our specific research questions were as follows: (1) Is telerehabilitation associated with improvement in pain and physical function in knee OA compared with traditional therapy or usual care? (2) Are different telerehabilitation strategies associated with improvement in pain and physical function in knee OA compared with traditional therapy or usual care?

## Methods

### Literature Search

This review was conducted according to the Cochrane Collaboration methodological guidelines [40]. We searched 6 databases (PubMed, Web of Science, EMBASE, Cochrane Library databases, CNKI, and WANFANG) for RCTs published from January 1, 2000, to September 3, 2023. Relevant articles and reference lists were manually searched. The obtained articles were reviewed by 2 investigators (WX and HX) independently.

### Search and Eligibility Criteria

The overall search strategies were performed by using a combination of relevant medical subject heading terms and free-text words (telemedicine or e-health or telehealth or telerehabilitation or internet or web or online or app or wearable or sensor) and knee OA. The detailed search strategy is described in Multimedia Appendix 1.

### Inclusion and Exclusion Criteria

#### Types of Trials

We included RCTs that were peer reviewed and written in English or Chinese. Clinical observations, reviews, case reports, conference papers, letters, abstracts, studies published in languages other than English and Chinese, and those with insufficient data were excluded.

#### Types of Participants

We included patients with knee OA, irrespective of age and the stage of pain.

#### Types of Interventions

We included unimodal intervention (telerehabilitation therapy alone) or multimodal intervention (telerehabilitation therapy in combination with other interventions). In this review, the scope of telerehabilitation was defined as “the use of information technology to monitor patients from a distance, including technologies such as telephone lines, broadband, or wireless networks” [41,42]. Participants in the control group could undergo other interventions (eg, interventions without telerehabilitation, standard treatment, or no intervention).

#### Types of Outcomes Measured

The primary outcomes were pain intensity and physical function.

### Data Extraction and Management

Data extraction was completed by 2 authors (WX and HX) independently. Disagreements between the 2 investigators were resolved by a third reviewer (BJJ). The extracted data included basic information of the study, participants, type of intervention for the experimental group and the control group, and outcomes. Outcomes reported as continuous variables are presented as the mean (SD).

### Telerehabilitation Strategies Extracted

We extracted 8 telerehabilitation strategies according to 3 categories: technology applications (1 strategy), care objectives (3 strategies), and care support methods (4 strategies) (Table 1).

**Table 1:** Extracted telerehabilitation strategies for the subgroup meta-analysis on telerehabilitation interventions for knee osteoarthritis.

Strategies		Descriptions
**Technology applications**		
	Mobile health system	A system was used in the telerehabilitation programs that involved a software app designed for mobile devices.
**Objectives**		
	Education	The telerehabilitation program included an objective involving knee osteoarthritis education via audio, animation, or text messages.
	Physiotherapy	Physiotherapy was monitored or assessed via an electronic device, thereby assisting participants in conducting exercises.
	Depression and anxiety	An objective was provided to address depression and anxiety in patients through the telerehabilitation program.
**Support methods**		
	Physician support	Physicians were included in the telerehabilitation program to provide clinical intervention.
	Physiotherapist support	Physiotherapists were included in the telerehabilitation program to provide clinical intervention.
	Psychologist support	Psychologists were included in the telerehabilitation program to provide clinical intervention.
	Monitoring symptoms	Automated systems were used to monitor the patients’ data and provide reminders and notifications to the patients.

### Review Outcomes

The primary outcome measures were focused on pain assessed by the Western Ontario and McMaster Universities Osteoarthritis (WOMAC) pain subscale and physical function assessed by WOMAC functional subscale.

### Risk of Bias

A summary of the methodological risk of bias of the included studies was conducted in accordance with the Cochrane Handbook for Systematic Reviews of Interventions [34] by 2 investigators (JYW, LJL) using the risk of bias tool in the Cochrane Collaboration’s review-writing software RevMan (version 5.4). The risk of bias assessment of RCTs mainly included 7 aspects: random sequence generation, allocation sequence concealment, blinding of participants and personnel, blinding of outcome assessment, completeness of outcome data, and selective outcome reporting [40]. Each item was judged as being at a high, low, or unclear risk of bias [40].

### Meta-Analysis

The mean (SD) of continuous outcome variables after therapy was used to calculate the total effect size via the mean difference and 95% CI. The standardized mean difference (SMD) was calculated when studies used different methods to measure the same outcome. The heterogeneity of RCTs in each group was examined by the *P* value and *I*^2^ statistic. A random-effects model was applied when *P*<.05 or *I*^2^>50%; otherwise, a fixed-effects model was used [43]. The meta-analysis methods and tests were performed using RevMan 5.4.

## Results

### Search Results

**Figure 1:**
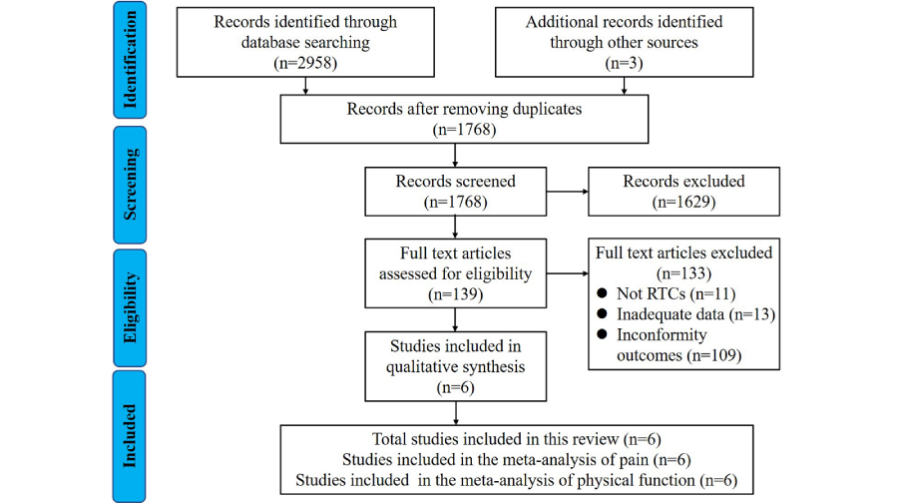
Flow diagram of the review. RTC: randomized controlled trial.

The literature search results are presented in Figure 1. A total of 2958 articles were searched from the databases and 3 articles were searched manually, resulting in a total of 2961 articles. After removing 1190 duplicate articles, 1768 articles were obtained for screening. Of these, 1629 articles were excluded for not fulfilling the inclusion criteria, and 139 articles were obtained for a full-text assessment. Of these, 133 articles were excluded based on the inclusion and exclusion criteria. Finally, 6 RCTs were included in this review, for which pain was assessed by the WOMAC pain subscale and physical function was assessed by WOMAC functional subscale.

**Figure 2:**
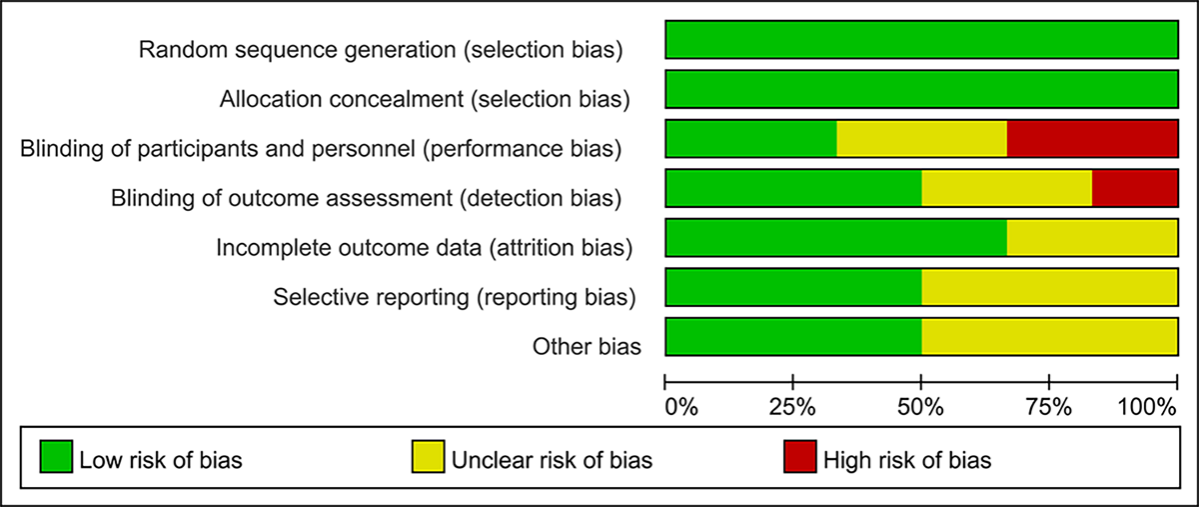
Risk of bias assessment. Judgments about each methodological quality item are presented as percentages.

**Figure 3:**
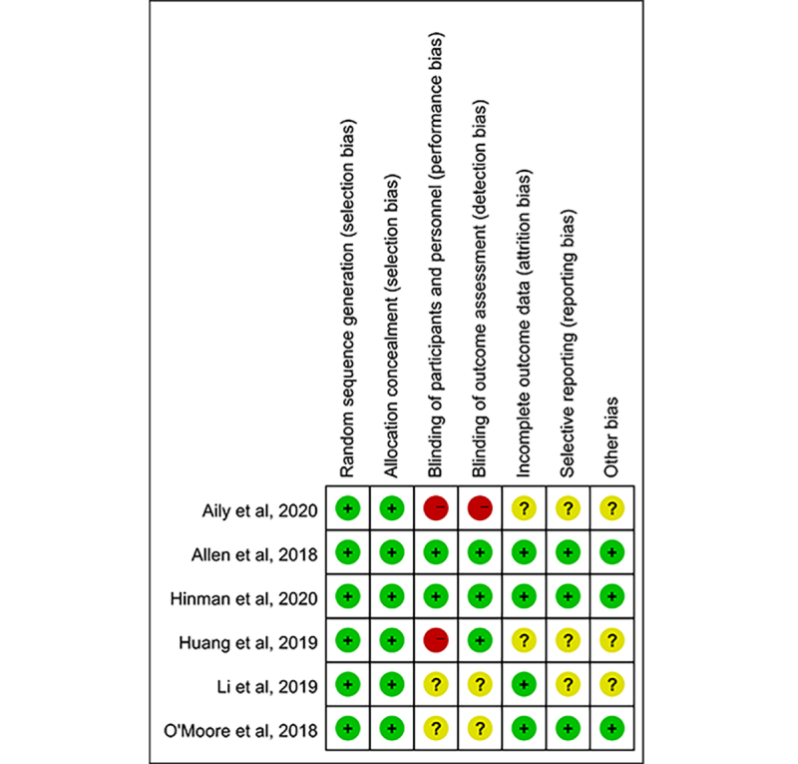
Risk of bias summary. Judgements about each risk of bias item were summarized for each included randomized controlled trial.

Among the bias risk assessment elements, the blinding of participants and personnel was the least used method in the RCTs (Figure 2). There were 2 RCTs that did not blind participants and personnel (Figure 3 [27-32]), while 2 RCTs did not report their blinding status and 2 RCTs used a blinding approach. Meanwhile, selective reporting and other bias was the least reported element, and 3 RCTs (50%) had an unclear risk of bias.

### Participant Characteristics

The 6 RCTs included 734 participants. The baseline descriptive characteristics (country, sample size, age, and gender) of the 6 studies included in the systematic review are summarized in Table 2. One study was from the United States [28], 2 were from Australia [27,32], 1 was from Brazil [31], and 2 were from China [29,30]. The mean age of patients with knee OA ranged from 53.1 (SD 8.5) years to 72.25 (SD 8.84) years, and all studies included both men and women.

**Table 2:** Baseline characteristics of studies in the systematic review.

Reference, country	Patient characteristics		Comparison	Intervention	Intervention time (weeks)	Outcome
	Participants, n (female/male)	Age (years), mean (SD)				
Allen et al, 2018 [28], United States	350 (251/99)	Group 1 (n=140): 65.7 (10.3); Group 2 (n=68): 64.3 (12.2); Group 3 (n=142): 65.3 (11.5)	Group 1: physiotherapy (evidence-based approach); Group 2: wait without any therapy	Group 3: internet-based exercise training	48	WOMAC^a^, 30-s chair stand, TUG^b^, 2-min step test, unilateral stand time
O'Moore et al, 2018 [27], Australia	69 (55/14)	Group 1 (n=25): 59.68 (6.01); Group 2 (n=44): 63.16 (7.38)	Group 1: treatment as usual	Group 2: iCBTe^c^ program for depression added to treatment as usual	10	PHQ-9^d^, K-10^e^, ASES^f^, WOMAC, SF-12^g^
Huang et al, 2019 [29], China	40 (30/10)	Group 1 (n=20): 72.25 (8.84); Group 2 (n=20): 67.25 (10.97)	Group 1: conventional therapy in the clinic	Group 2: conventional therapy plus a brief GOH^h^-based intervention	24	WOMAC; drop-out rates; MFI^i^; HADS^j^; PSQI^k^
Li et al, 2019 [30], China	80 (60/20)	Group 1 (n=25): 59.11 (9.13); Group 2 (n=26): 61.71 (9.58); Group 3 (n=29): 59.24 (15.43)	Group 1: electro-acupuncture and moxibustion in hospital; Group 2: percutaneous electrical acupoint stimulation therapy in hospital	Group 3: transcutaneous electrical acupoint stimulation therapy through the Anrui app	1	VAS^l^, WOMAC; 30-s chair stand test, 40-m fast paced walk test, stair climb test
Hinman et al, 2020 [32], Australia	175 (110/65)	Group 1 (n=88): 62.5 (8.1); Group 2 (n=87): 62.4 (9.1)	Group 1: existing service from the Musculoskeletal Help Line	Group 2: same exercise protocol as existing service group and consultations with a physiotherapist	48	NRS^m^, WOMAC, cost-effectiveness
Aily et al, 2020 [31], Brazil	20 (10/10)	Group (n=10): 54.8 (8.3); Group 2 (n=10): 53.1 (8.5)	Group 1: supervised periodized circuit training 3 times a week	Group 2: same exercise protocol following the orientations to the exercises through videos, and they received periodic telephone calls	14	VAS, WOMAC, 30-s chair stand test, 40-m fast paced walk test, stair climb test

^a^WOMAC: Western Ontario and McMaster Universities Osteoarthritis index.

^b^TUG: time up and go.

^c^iCBTe: internet-based cognitive-behavioral therapy.

^d^PHQ-9: 9-item patient health questionnaire.

^e^K-10: Kessler-10.

^f^ASES: arthritis self-efficacy scale.

^g^SF-12: short form 12-item.

^h^GOH: Guangdong Online Hospital.

^i^MFI: multidimensional fatigue inventory.

^j^HADS: hospital anxiety and depression scale.

^k^PSQI: Pittsburgh sleep quality index.

^l^VAS: visual analog scale.

^m^NRS: numeric rating scale.

### Telerehabilitation Strategies

We extracted 9 telerehabilitation strategies from the 6 RCTs, as shown in Table 3. Some strategies were commonly used, such as physiotherapy support (n=4, 67%), physician support (n=4, 67%), intervention for education (n=3, 50%), and physiotherapist support (n=3, 50%). Strategies that were not commonly used included intervention for depression and anxiety (n=2, 33%) and psychologist support (n=2, 33%). The telerehabilitation programs in the RCTs generally contained multiple strategies, with a mean of 4.33 strategies per care program.

**Table 3:** Telerehabilitation strategies and randomized controlled trials (RTCs) included in the meta-analysis. A binary scoring system was used (0=no and 1=yes). All RTCs used a mobile health system.

Reference	Participants, n	Objectives				Support methods			
		Education	Physiotherapy	Depression and anxiety	Physician		Physiotherapist	Psychologist	Monitoring
[27]	69	1	0	1	0		0	1	0
[28]	350	0	1	0	1		1	0	1
[29]	40	1	0	1	1		0	1	0
[30]	80	0	1	0	1		0	0	0
[32]	175	1	1	0	0		1	0	0
[31]	20	0	1	0	1		1	0	1
Total	734	3	4	2	4		3	2	2

### Overall Effectiveness of Telerehabilitation

**Figure 4:**
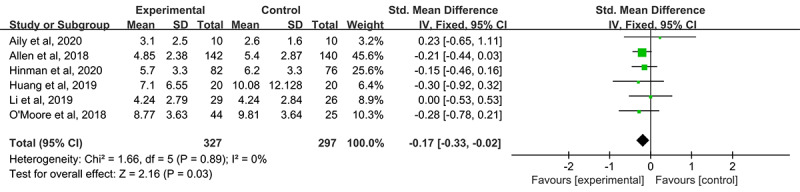
Forest plot of the included studies comparing the effect of telerehabilitation and conventional treatment on pain according to the Western Ontario and McMaster Universities Osteoarthritis index pain subscale.

**Figure 5:**
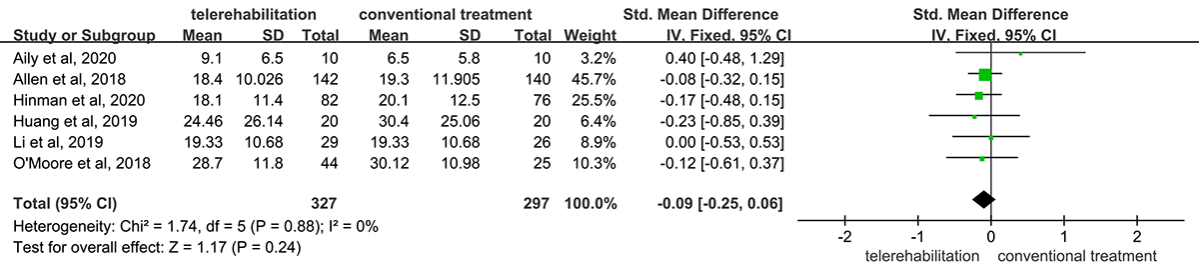
Forest plot of the included studies comparing the effect of telerehabilitation and conventional treatment on physical function based on the Western Ontario and McMaster Universities Osteoarthritis index function subscale.

We assessed pain and physical function in the 6 RCTs (n=624) using the WOMAC pain and function subscales, respectively. The outcomes of pain and function with 95% CIs are shown in Figure 4 and Figure 5, respectively [27-32]. Overall, telerehabilitation was found to be more effective than conventional treatment for the improvement of pain (SMD –0.21, 95% CI –0.35 to –0.07; *P*=.003), but not physical function (SMD –0.09, 95% CI –0.25 to 0.06; *P*=.24). The outcomes of both pain and physical function were heterogeneous, with a low level of heterogeneity (*I*^2^=0%) in both the pain and physical function outcomes.

### Comparison of Different Telerehabilitation Strategies

Compared with conventional treatment, the group of RCTs that provided various telerehabilitation strategies was not found to be more effective for improving pain and physical function, as shown in Tables 4 and 5.

**Table 4:** The effect of telerehabilitation strategies on pain for randomized controlled trials (RCTs) that applied the strategy in the telerehabilitation intervention.

Strategies			RCTs, n (n participants)		Effect					Heterogeneity				
					SMD^a^ (95% CI)		*P* value		Chi-square (*df*)			*P* value		*I*^2^ (%)
**Objectives**														
	Education	3 (284)		–0.21 (–0.45 to 0.04)		.10		0.30 (2)			.86		0	
	Physiotherapy	4 (625)		–0.15 (–0.32 to 0.02)		.09		1.25 (3)			.74		0	
	Depression and anxiety	2 (109)		–0.29 (–0.68 to 0.10)		.14		0.00 (1)			.97		0	
**Support methods**														
	Physician	4 (490)		–0.17 (–0.36 to 0.03)		.10		1.45 (3)			.69		0	
	Physiotherapist	3 (545)		–0.15 (–0.36 to –0.06)		.15		1.25 (2)			.53		0	
	Psychologist	2 (109)		–0.29 (–0.68 to 0.10)		.14		0.00 (1)			.97		0	
	Monitoring symptoms	2 (370)		–0.18 (–0.41 to 0.05)		.12		0.88 (1)			.35		0	

^a^SMD: standardized mean difference.

**Table 5:** The effect of telerehabilitation strategies on physical function for randomized controlled trials (RCTs) that applied the strategy in the telerehabilitation intervention.

Strategies		RCTs, n (n participants)	Effect		Heterogeneity		
			SMD^a^ (95% CI)	*P* value	Chi-square (*df*)	*P* value	*I*^2^ (%)

**Objectives**							
	Education	3 (284)	–0.17 (–0.41 to –0.08)	.18	0.07 (2)	.97	0
	Physiotherapy	4 (625)	–0.08 (–0.25 to –0.09)	.36	1.53 (3)	.68	0
	Depression and anxiety	2 (109)	–0.16 (–0.55 to 0.22)	.41	0.07 (1)	.79	0
**Support methods**							
	Physician	4 (490)	–0.06 (–0.26 to 0.14)	.54	1.41 (3)	.70	0
	Physiotherapist	3 (545)	–0.09 (–0.27 to –0.09)	.33	1.43 (2)	.49	0
	Psychologist	2 (109)	–0.16 (–0.55 to 0.22)	.41	0.07 (1)	.79	0
	Monitoring symptoms	2 (370)	–0.05 (–0.28 to 0.18)	.66	1.08 (1)	.30	7

^a^SMD: standardized mean difference.

## Discussion

### Principal Findings

This systematic review and meta-analysis investigated whether pain and physical function in patients with knee OA could be improved by telerehabilitation programs and different telerehabilitation strategies. The results showed that the pain, but not the physical function, of patients with knee OA could be significantly improved by telerehabilitation compared with traditional therapy or usual care. Subgroup analyses revealed that the pain and physical function in patients with knee OA could not be further improved by combining different telerehabilitation strategies. This finding adds evidence to support telerehabilitation interventions for patients with knee OA.

### Relationship With Previously Published Literature

Pain is the primary symptom in patients with knee OA; it occurs gradually, worsens with time, can lead to many problems, and is the number 1 reason most patients seek medical attention. Consistent with previous systematic reviews and meta-analyses [44-48], our findings indicated that the pain of knee OA could be relieved by telerehabilitation after patients are discharged from the hospital. The positive effects may benefit from telehealth intervention features, which enable patients living in remote or medically resource-poor areas to receive professional medical help [26]. Programs such as IBET were shown to be effective for pain reduction [22], which may be attributed to personalized exercise plans to reduce pain [22]. Meanwhile, telerehabilitation strategies, including educational lectures, medical suggestions, and psychotherapy were effective for the reduction of pain [23]. Furthermore, Bennell et al [49] suggested that telehealth-delivered exercise and diet programs improved pain in people with knee OA and overweight or obesity, which indicated that diet also plays an important role in alleviating pain in patients with knee OA. These telerehabilitation programs could combine various interventions to ease pain. For patients, the place of rehabilitation exercise is more convenient.

As for the effect of telerehabilitation on physical function, this systematic review and meta-analysis suggested that the physical function of patients with knee OA could not be improved by telerehabilitation. Previous systematic reviews have reported inconsistent results [22,44-48,50]. Some studies [22,44-46,50] showed no significant improvement in physical function in patients with knee OA. For example, Allen et al [22] found that there was no significant difference in the effect of network sports training on improving physical function in patients with knee OA compared to the conventional physical therapy group, which may be related to the emphasis on exercise training guidance and low patient participation. However, Safari et al [47] found that a digital-based, structured self-management program improved physical function in patients with knee OA, and similar results were reported by Schäfer et al [48]. There was no significant improvement in physical function following internet-based exercise training compared with face-to-face supervised exercise [22]. Hinman et al [26] showed that physical function could be modestly improved by telephone-delivered physiotherapist-led exercise advice and support at 6 months, but functional benefits were not sustained at 12 months. The reasons for this result might be due to the fact that the recruited participants often had better baseline physical function and functional improvement required a longer-term intervention and more intervention forms.

In addition to performing a meta-analysis of overall effectiveness, we used a subgroup comparison method to analyze the effects of different telerehabilitation strategies on the improvement of pain and physical function. The results indicated that there is no correlation between different telerehabilitation strategies and the improvement of pain and physical function in patients with knee OA. Anwer et al [51] found that home exercise programs with and without supervised clinic-based exercises were beneficial in the management of knee OA, which is consistent with the results of this review. However, Sinatti et al [52] showed that education seems to be effective in reducing pain and improving function in patients with knee OA. We speculate that this may be related to the sample size included in the various intervention strategies and the length of intervention time. However, these strategies should not be ignored, and further investigation of their contribution to knee OA treatment remains important to continuously improve telerehabilitation outcomes in future studies.

### Limitations

There are several limitations in this systematic review and meta-analysis. First, fewer studies were included in this review. Second, the objective of our systematic review was to evaluate different telerehabilitation strategies, and our meta-analysis did not rigorously exclude RCTs with risk of bias. Third, the outcome measures to assess pain and physical function in patients with knee OA were subjective. Fourth, moderator variables (eg, age, gender, and sample size) for telerehabilitation effects were not analyzed. Finally, considering the diversity of outcome indicators, only studies using the WOMAC scale were included in the analysis to ensure the reliability of the study.

### Conclusions

Internet-based rehabilitation is a promising strategy for patients with knee OA. Compared with conventional rehabilitation, the results of this meta-analysis suggest that telerehabilitation programs could improve the pain but not the physical function of patients with knee OA. Meanwhile, there was no significant correlation between different telerehabilitation strategies and the pain and physical function of patients with knee OA. These results indicate that telerehabilitation is beneficial for the implementation of home rehabilitation exercises for patients with knee OA, thereby reducing the economic burden of health. However, there is currently relatively little research on the effects of telerehabilitation on knee OA. In the future, more high-quality studies with large samples are needed to focus on the long-term outcomes of telerehabilitation for patients with knee OA and the effect of different telerehabilitation strategies. The completion of high-quality trials will ultimately advance our knowledge about optimal telerehabilitation strategies for patients with knee OA.

## Appendix

Multimedia Appendix 1 Search strategies.

Multimedia Appendix 2 PRISMA Checklist.

## Abbreviations

OA: osteoarthritis
RCT: randomized controlled trial
SMD: standardized mean difference
WOMAC: Western Ontario and McMaster Universities Osteoarthritis index

## Data Availability

All data generated or analyzed during this study are included in this published article. Further inquiries can be directed to the corresponding author.
