# Supplementary material for: Effectiveness of Different Telerehabilitation Strategies on Pain and Physical Function in Patients With Knee Osteoarthritis: Systematic Review and Meta-Analysis
Source: J Med Internet Res. 2023 Dec 4;25:e40735. doi: 10.2196/40735 (PMC10728785; doi:10.2196/40735)
Supplement: Multimedia Appendix 1 [file jmir_v25i1e40735_app1.docx]

Multimedia Appendix 1: Search Strategy

Database: Pubmed <2000 to 2023>

Date searched: September 4, 2023

Search Strategy:

--------------------------------------------------------------------------------

1 ("Telemedicine"[MeSH Terms]) OR ("Telemedicine"[Title/Abstract]) OR ("Telerehabilitation"[Title/Abstract]) OR ("E-health"[Title/Abstract]) OR ("Telehealth"[Title/Abstract]) (64002)

2 ("Internet"[MeSH Terms]) OR ("Internet"[ Title/Abstract]) OR ("Internet-Based Intervention"[Title/Abstract]) OR ("Web"[Title/Abstract]) OR ("Online"[Title/Abstract]) OR ("App"[Title/Abstract]) OR ("Wearable"[ Title/Abstract]) OR ("Sensor"[ Title/Abstract]) (693632)

3 "knee osteoarthritis"[ Title/Abstract] (16373)

4 1 or 2 (739706)

5 3 and 4 (828)

Database: Web of Science <2000 to 2023>

Date searched: September 4, 2023

Search Strategy:

--------------------------------------------------------------------------------

1. TS = “Telemedicine” OR “Telerehabilitation” OR “E-health” OR “Telehealth” (57464)

2 TS = “Internet” OR “Internet-Based Intervention” OR “Web” OR “Online” OR “App” OR “Wearable” OR “Sensor” (2266169)

3 TS = “knee osteoarthritis” (29744)

4 1 or 2 (2308332)

5 3 and 4 (1239)

Database: EMBASE <2000 to 2023>

Date searched: September 4, 2023

Search Strategy:

--------------------------------------------------------------------------------

1 ‘Telemedicine’ OR ‘Telerehabilitation’ OR ‘E-health’ OR ‘Telehealth’ OR ‘Internet’ OR ‘Internet-Based Intervention’ OR ‘Web’ OR ‘Online’ OR ‘App’ OR ‘Wearable’ OR ‘Sensor’ (1348263)

1. ‘exp knee osteoarthritis’ or ‘knee osteoarthrosis’ or ‘knee cartilage’ or ‘degenerative arthritis’ (4645)

3 1 and 2 (131)

Database: Cochrane Library <2000 to 2023>

Date searched: September 4, 2023

Search Strategy:

--------------------------------------------------------------------------------

1 Title Abstract Keyword = (Telemedicine OR Telerehabilitation OR E-health or Telehealth OR Internet OR Internet-Based Intervention OR Web OR Online OR App OR Wearable OR Sensor) (62172)

2 Title Abstract Keyword = (knee osteoarthritis) (16353)

1. 1 and 2 (621)

Database: China national knowledge infrastructure (CNKI)

Date searched: September 4, 2023

Search Strategy:

--------------------------------------------------------------------------------

1 SU='远程康复' OR SU='远程医疗' OR SU='互联网' OR SU='微信' OR SU='QQ'(576453)

2 SU='膝骨关节炎' OR SU='关节炎' OR SU='膝关节炎' (117863)

3 1 and 2 (81)

Database: WANFANG Data

Date searched: September 4, 2023

Search Strategy:

--------------------------------------------------------------------------------

1 题名或关键词:(远程康复 ) or 题名或关键词:(远程医疗 ) or 题名或关键词:(互联网 ) or 题名或关键词:(移动医疗 ) or 题名或关键词:(微信) or 题名或关键词:(QQ) (25226)

2 (题名或关键词:(膝骨关节炎) or 题名或关键词:(关节炎) or 题名或关键词:(膝关节炎)) (117051)

1. 1 and 2 (58)
